# Influence of endodontic sealers artifacts in the detection of vertical root fractures

**DOI:** 10.1590/0103-6440202204392

**Published:** 2022-03-07

**Authors:** Marcelo Gusmão Paraiso Cavalcanti, Fernanda Cristina Salineiro, Fabiana Mesquita Barros, Francisco Barbara Abreu Barros

**Affiliations:** 1 Department of Stomatology, School of Dentistry, Universidade de São Paulo, São Paulo, SP, Brazil.

**Keywords:** Vertical root fractures, artifact, cone-beam computed tomography, endodontic sealers

## Abstract

The aim of this study was to compare the influence of endodontic sealers artifacts on the detection of vertical root fracture in cone beam computed tomography (CBCT). Premolars and central incisors were assigned into five different groups: Control, Pulp Canal Sealer, AH Plus, Sealer 26, and BC Sealer (n= 10, per group). VRFs were mechanically induced and the teeth were inserted into an image phantom. Subsequently, CBCT (Cranex 3Dx, Soredex, Tuusula, Finland) images were obtained and two observers were asked separately to identify root fracture, by visual analysis. For both premolar and central incisors, kappa coefficients of intraobserver agreement varied from good to excellent (K: 80% - 87%), and the values for interobserver agreement varied from fair to moderate (K: 30% - 35%). As follows, the area under the curve (AUC) of receiver operating characteristic (ROC) values for VRFs highlighted that the use of BC sealer reduced the observers’ ability to discriminate VRFs relative to other sealers. Moreover, sensitivity values for premolars teeth ranged from 20% to 60%, and specificity ranged from 60% to 100%; while sensitivity values for central incisors ranged from 30% to 70%, and specificity ranged from 70% to 100%. In conclusion, the low sensitivity values, mainly for premolars, demonstrated the difficulty in VRF diagnosis. Furthermore, BC Sealer induced significantly more imaging artifacts than other sealers. These results highlighting that endodontic sealers may interfere with the diagnosis of VRFs.

## Resumo

O objetivo deste estudo foi comparar a influência dos artefatos de cimentos endodônticos na detecção de fratura radicular vertical (FRV) em tomografia computadorizada de feixe cônico (TCFC). Pré-molares e incisivos centrais foram divididos em cinco grupos diferentes: Controle, Pulp Canal Sealer, AH Plus, Sealer 26 e BC Sealer (n = 10, por grupo). As FRVs foram induzidas mecanicamente e os dentes inseridos em uma réplica da mandibula humana. Posteriormente, imagens de TCFC (Cranex 3Dx, Soredex, Tuusula, Finlândia) foram obtidas e dois observadores foram solicitados a identificar fratura radicular separadamente, o critério utilizado foi a análise visual. Tanto para pré-molares quanto para incisivos centrais, os coeficientes kappa de concordância intraobservador variaram de bom a excelente (K: 80% - 87%), e os valores de concordância interobservador variaram de regular a moderado (K: 30% - 35%). A seguir, a área sob a curva (AUC) dos valores da característica de operação do receptor (ROC) para FRVs destacou que o uso do cimento BC Sealer reduziu a capacidade dos observadores de discriminar FRVs em relação a outros cimentos endodônticos. Além disso, os valores de sensibilidade para pré-molares variaram de 20% a 60%, e a especificidade variou de 60% a 100%; enquanto os valores de sensibilidade para incisivos centrais variaram de 30% a 70%, e a especificidade variou de 70% a 100%. Em conclusão, os baixos valores de sensibilidade, principalmente para pré-molares, demonstraram uma dificuldade no diagnóstico de FRV. Além disso, o BC Sealer induziu significativamente mais artefatos de imagem do que outros cimentos. Esses resultados evidenciam que os cimentos endodônticos podem interferir no diagnóstico das FRVs.

## Introduction

Vertical root fractures (VRFs) can occur during or after root canal treatment, being the most severe type of longitudinal fracture. This type of fracture usually originates from the root of the tooth and extends to the coronal direction ^1^ ^,^ ^2^ ^,^ ^3^. In line with the need to assess the extent of the VRFs, cone-beam computed tomography (CBCT) provides greater three-dimensional precision in images, presenting real information about the size, shape, texture, and adjacent structures of teeth ^3^ ^,^ ^4^. For the dental surgeon, the diagnosis of CBCT imaging can be challenging due the presence of artifacts generated by radiopaque materials, which can cause image distortions in the form of streaks, shadows orientated along the projection lines, line structures, and even imitating fracture gaps. Therefore, these issues complicate the diagnosis of root fractures ^3^ ^,^ ^5^ ^,^ ^6^ ^,^ ^7^ ^,^ ^8^ ^,^ ^9^ ^,^ ^10^. Artifacts may occur due to differences in attenuation and absorption of X-rays beams by high-density materials, such as metallic posts and root canal fillers. The beam hardening appears due to the substantial absorption of lower energetic rays by an object with higher atomic number and, consequently, there is an increase of the medium energy of the beam ^7^ ^,^ ^9^ ^,^ ^11^. Some materials that cause these distortions are endodontic filling materials, such as gutta-percha and endodontic sealers used during the filling of the root canal. It is known that commercially available gutta-percha cones are composed of inorganic (zinc oxide and metallic sulfates) and organic compounds (waxes, resins, and gutta-percha). Thus, the opacity noticed by CBCT is directly related to the proportion of these materials ^10^ ^,^ ^12^. Endodontic sealers, on the other hand, may lead to differences in density, since they have the presence of radiopacifiers and other chemical substances in their formulations, such as zinc oxide, bismuth subcarbonate, bismuth oxide, and barium sulfate ^13^. However, each endodontic sealer bears its purpose and its differential according to its formulation. Many studies analyzed mechanical strength, resistance to root fracture, biocompatibility, adhesion, and other factors ^14^ ^,^ ^15^. In order to cover different compositions of sealers, the materials for the current study were selected considering biocompatibility, better adhesion to dentin, and resistance to fracture. Currently, few studies investigate the influence of endodontic material in radiological diagnosis. Therefore, this study aimed to compare the influence of four endodontic sealers in the production of artifacts in CBCT imaging for VRFs detection in central incisor and premolar teeth.

## Material and methods

### Preparation of Samples

The present study was approved by the ethics committee of our institution, under the protocol 1.121.863.

Single-rooted premolar (n = 100) and central incisor (n=100) teeth were selected for this study. The teeth were scanned using CBCT (Cranex 3Dx, Soredex, Tuusula, Finland) to exclude samples with root resorption, larger restorations (beyond the cementoenamel junction), cracks, fractures, more than one root canal or dilacerations. One operator prepared all teeth for the study and executed the CBCT imaging exams. The anatomic crowns of the selected teeth were sectioned at the cementoenamel junction, using a carborundum disc propelled by an air turbine (KaVo Dental, Biberach, Baden-Württemberg, Germany). To perform the endodontic instrumentation, an endodontist used the Easy ProDesign (Easy equipment, Belo Horizonte, MG, Brazil) rotatory instrument up to size #.25/06 and #.25/08.

Premolar and central incisor teeth were assigned in five different groups:

- Control: instrumented root without endodontic filling;
- Pulp Canal Sealer Group: instrumented root with conventional gutta-percha and Pulp Canal Sealer;
- AH Plus Group: instrumented root with conventional gutta-percha and AH Plus sealer;
- Sealer 26 Group: instrumented root with conventional gutta-percha and Sealer 26;
- BC Sealer Group: instrumented root with bioceramic gutta-percha and BC Sealer.

The obturation was performed with a single conventional gutta-percha point at working length for all groups according to the manufacturer's instructions. Instead of conventional gutta-percha, the bioceramic gutta-percha points were employed for the BC Sealer group. The composition and manufacturers of the root canal sealers was displayed in Table 1.

**Table 1:** The components and manufacturers of root canal sealers used in the study

	Composition	Manufacturer
Pulp Canal Sealer	Powder: zinc oxide, polymeric resin, silver	Kerr Corporation, Orange, CA, U.S.A.
	Liquid: eugenol, balm from Canada	
AH Plus	Paste A: epoxy resin, calcium tungstate, zirconium oxide, aerosil, iron oxide	Dentsply, Petrópolis, RJ, Brazil
	Paste B: Adamantane amine N, N’-dibenzyl-5oxanonane diamine-1,9, TCD-diamine calcium tungstate, zirconium oxide, aerosil, silicone oil	
Sealer 26	Powder: Bismuth Trioxide, Calcium Hydroxide, hexamethylenetetramine, Titanium Dioxide	Dentsply, Petrópolis, RJ, Brazil
	Paste: bisphenol epoxy resin	
BC Sealer	zirconium oxide, tricalcium silicate, dicalcium silicate, calcium hydroxide and the gutta-percha used in this system is coated with bioceramic nanoparticles	Angelus, Londrina, PR, Brazil

### Fracture induction

Ten teeth were selected from each group and induced the root fractures. An operator that was not involved in the CBCT imaging analyses or the endodontic instrumentation handled the simulation of root fractures. The tooth was positioned in a horizontal plane fixed by a bench vise and induced the root fracture through mechanical force exerted gradually by a nail with a chisel-shaped tip ^13^ ^,^ ^16^. The mechanical force was directed along the root axis to induce a vertical fracture. The two fragments obtained were joined with the use of Super Bonder cyanoacrylate adhesive ^2^ ^,^ ^6^ ^,^ ^7^ (Henkel Brazil, Loctite, São Paulo). Roots that fragmented into more than two parts were excluded from the sample according to previous studies ^8^ ^,^ ^9^ ^,^ ^11^.

### Image Acquisition

After endodontic treatment and fracture of the selected specimens, the premolars and incisors were inserted into an image phantom, fabricated with dental stone (Durone, Dentsply, York, Pennsylvania, EUA) and perforated using water-cooled diamond burs driven by an air turbine (300,000 rpm), at the anterior and posterior regions to simulate the alveolar sockets and the position of the teeth in the human mandible. Premolar and central incisor teeth were inserted at the anterior and posterior perforations, respectively. In order to simulate the attenuation of the x-rays beams caused by soft tissues, the mandible model was placed in a cylindrical plastic container filled with water so it remained fully submerged at the time of the tomographic acquisition ^17^. Fifty CBCT (Cranex 3Dx, Soredex, Tuusula, Finland) scans were performed adjusted at field of view (FOV) of 8 x 6 cm, voxel size of 0.15 mm, acquisition time of 15 seconds, 90 kVp, and 10 mA - Hight Definition Protocol ^6^ ^,^ ^7^ ^,^ ^9^.

### CBCT Imaging Evaluation

**Figure 1:**
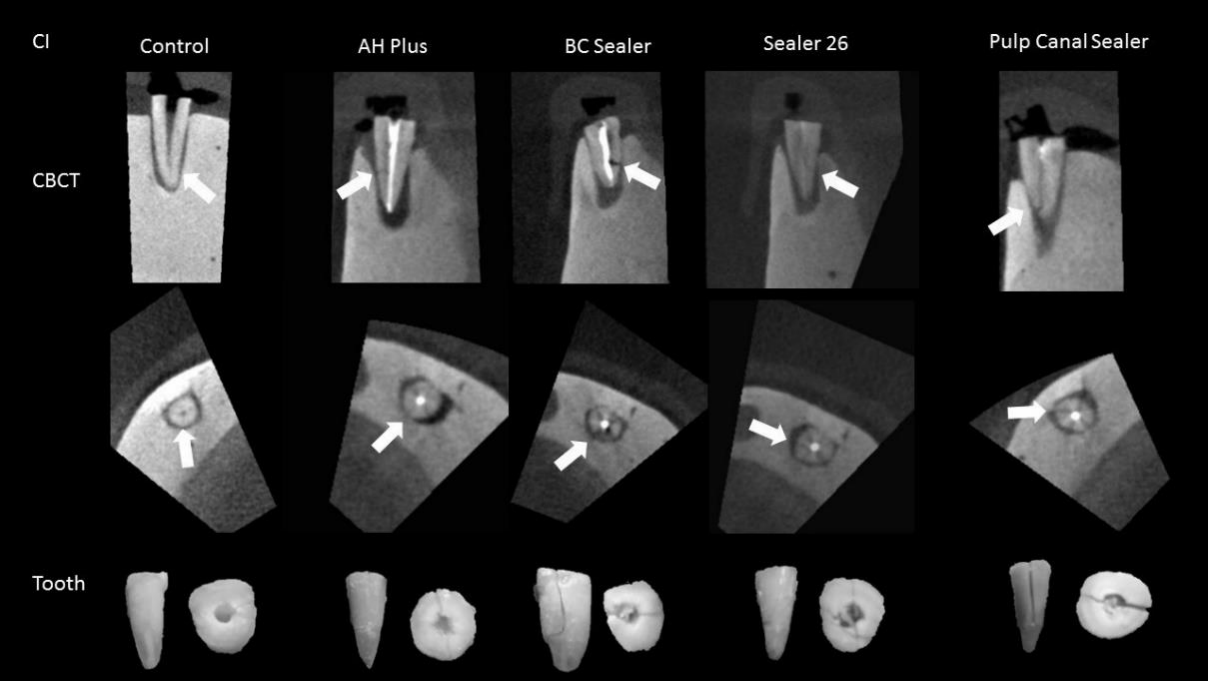
Central incisors - CBCT images of control and test groups on the first line (sagittal reconstruction) and second line (axial reconstruction). The third line is macroscopy images of the teeth. The white arrow indicates the hypodense line of root fracture.

**Figure 2:**
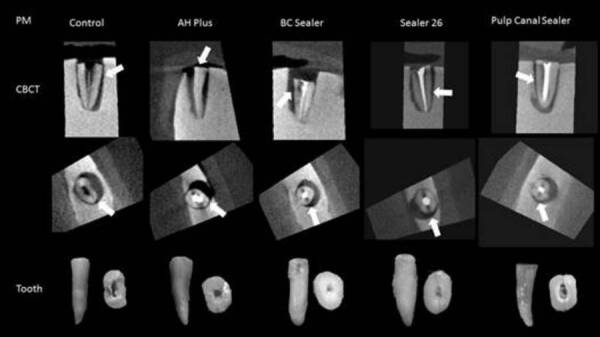
Premolars - CBCT images of control and test groups on the first line (sagittal reconstruction) and second line (axial reconstruction). The third line is macroscopy images of the teeth. The white arrow indicates the hypodense line of root fracture.

All CBCT images were exported as Digital Imaging and Communication in Medicine (DICOM) files and imported to a workstation (iMac 27”, Apple, Cupertino, CA, USA). A DICOM viewer software (OsiriX MD 1.2 64-bit, Pixmeo, Geneva, Switzerland) was employed to assess the images. Observation sequences for CBCT images were randomized through a website (www.random.org, Randomness and Integrity Services Ltd, Dublin, Ireland). Two blind previously calibrated and CBCT trained oral and maxillofacial radiologists, used the same workstation independently to perform the analyses. In order to assess intraobserver agreement, all images were evaluated after a two-week break. The teeth sequence used for the examiners was determined by software (Randomness 1.5.2, Andrew Merenbach, Los Angeles, CA, EUA). The criterion for the analysis was as follows: CBCT versus gold standard (direct visual analysis of the teeth) (Figures 1 and 2). The presence or absence of fracture was ranked with the aid of a 5-point Likert scale (1, definitely absent; 2, probably absent; 3, uncertain; 4, probably present; 5, definitely present) ^3^ ^,^ ^16^ ^,^ ^18^.

### Statistical analysis

Interobserver and intraobserver agreements were calculated with the use of the Kappa coefficients (slight agreement, 0.0-0.20; fair agreement, 0.21-0.40; moderate agreement, 0.41-0.60; good agreement, 0.60-0.80; and excellent agreement, 0.81-1.00) ^19^. The responses were tabulated with the criterion standard (direct visual analysis of the teeth) and plotted into a web-based calculator for receiver operating characteristic (ROC) curves (Russell H. Morgan, Department of Radiology and Radiologic Science, Johns Hopkins University, Baltimore, MD) ^3^ ^,^ ^20^ ^,^ ^21^. The ROC analysis was performed by observer responses, and the sensitivity and specificity values were calculated. The sensitivity and specificity were compared using the analysis of variance (ANOVA), with Tukey post-hoc test, adopting a 5% significance level, at p ≤ 0.05 (MedCalc software for Windows v.18, MedCalc Software, Ostend, Belgium). When the null hypothesis is accepted, it is assumed that there is no statistical difference between endodontic sealers.

## Results

Both premolar and central incisors kappa coefficients of intraobserver agreement varied from good to excellent (K: 80% - 87%), while the kappa values for interobserver agreement varied from fair to moderate (K: 30% - 35%) (Table 2). The AUC values derived from the ROC analysis of root fractures informs the diagnostic performance of independent observers in each group, which yielded a statistically significant difference between the BC Sealer performance compared to other sealers (Table 3). This finding highlights that BC Sealer restorations reduce the observers’ ability to discriminate VRFs relative to other sealers. Correspondingly, BC Sealer endodontic restoration for premolar and central incisor teeth also results in lower sensitivity scores (Table 4). Finally, the p value for sensitivity was 0.239 and the value for specificity was 0.039, but with Tukey post-hoc test no statistical difference was found. Sensitivity values for premolars teeth ranged from 20% to 60%, and specificity ranged from 60% to 100%; while sensitivity values for central incisors ranged from 30% to 70%, and specificity ranged from 70% to 100%. That is, low sensitivity values observed in the study, especially for premolars, acknowledges the difficulty in VRF diagnosis.

**Table 2:** Kappa coefficients of inter and intra-observer agreement for the subjective analysis of premolar (PM) and central incisors (CI).

	Obs. 1.1 x Obs 1.2	Obs. 2.1 x Obs 2.2	Obs. 1.1 x Obs 2.1	Obs. 1.2 x Obs 2.2
PM	83%	82%	32%	35%
CI	87%	80%	30%	32%

**Table 3:** Area under the curve (AUC) values derived from the ROC analysis of root fractures divided by test group for premolars (PM) and central incisors (CI). Two observers in each group.

	AUC (SD)	Control	Pulp Canal Sealer	AH Plus	Sealer 26	BC Sealer
PM	Observer 1	0.650 ^a^	0.645 ^a^	0.715 ^a^	0.530^a^	0.610^b^
		(0.121)	(0.119)	(0.116)	(0.120)	(0.133)
	Observer 2	0.635 ^a^	0.650 ^a^	0.645 ^a^	0.650 ^a^	0.590^b^
		(0.121)	(0.121)	(0.136)	(0.121)	(0.128)
CI	Observer 1	0.630^a^	0.745^a^	0.585^a^	0.655^a^	0.575^b^
		(0.122)	(0.102)	(0.135)	(0.124)	(0.118)
	Observer 2	0.745^a^	0.715^a^	0.710^a^	0.645^a^	0.535^b^
		(0.102)	(0.124)	(0.118)	(0.123)	(0.133)

(SD) standard deviation; Different letters = Statistical difference

**Table 4:** Sensitivity (Se) and Specificity (Sp) values of root fracture analysis in premolars (PM) and central incisors (CI).

		Control		Pulp Canal Sealer		AH Plus		Sealer 26		BC Sealer	
		Se	Sp	Se	Sp	Se	Sp	Se	Sp	Se	Sp
PM	Observer 1	60%	80%	40%	90%	50%	60%	30%	100%	20%	90%
	Observer 2	70%	70%	60%	60%	40%	70%	40%	90%	40%	80%
CI	Observer 1	70%	70%	60%	70%	50%	100%	40%	90%	30%	80%
	Observer 2	70%	90%	70%	90%	70%	80%	70%	80%	30%	90%

There were no statistical differences among the groups considering sensitivities or specificities (p > 0.05 through ANOVA)

## Discussion

Periapical radiographs are often employed to diagnose VRFs, which are represented by an association of halo-shaped radiolucency and angular bone loss ^5^. Since this imaging modality its bidimensional nature, it can be in some cases not conclusive for clinical diagnosis, CBCT provides three-dimensional images with more accurate information ^2^ ^,^ ^5^ ^,^ ^22^ ^,^ ^23^. Nevertheless, imaging artifacts in CBCT scans may mask fracture lines. In CBCT imaging, artifacts may occur due to differences in attenuation and absorption of x-rays beams by high-density materials. For instance, physical properties such as density of the enamel or materials used in oral rehabilitation and, secondarily, its location in the arch causes image distortions in the form of streaks, line structures, and even imitating fracture gaps. Furthermore, gutta-percha cones and metallic posts induce the presence of artifacts in CBCT images, complicating the interpretation of the test. Imaging artifacts decrease test sensitivity by mimicking fracture lines that may not exist and affects specificity as they overlap some root regions, making it difficult to visualize the fracture line ^1^ ^,^ ^7^ ^,^ ^8^ ^,^ ^9^ ^,^ ^10^ ^,^ ^11^ ^,^ ^16^ ^,^ ^24^. Overall, the current study aimed to compare the influence of four endodontic sealers in the production of imaging artifacts in CBCT scans for VRFs detection. 

Radiopaque materials, like gutta-percha, generates artifacts in CBCT scans. Gutta-percha cones are composed of organic (waxes, resins, and gutta-percha) and inorganic (barium sulfate and zinc oxide) materials. Thus, gutta-percha radiographic opacity depends on the proportion of organic to inorganic material ratio, which directly relates to gutta-percha’s radiopacity and its artifact formation ^10^ ^,^ ^12^. Hassan et al. ^11^ observed that the presence of gutta-percha significantly reduced specificity and did not significantly influence the sensitivity of CBCT exams. Likewise, Menezes et al. ^1^ concluded that the presence of gutta-percha in the root canals reduced the sensitivity and accuracy of the tomographic examination in the detection of VRFs. Both observed that the unrestored group had a significantly higher precision value when compared to the gutta-percha group. Salineiro et al. ^9^ further considered the difference in artifacts production by conventional gutta-percha and bioceramics restorations and concluded that both materials produced similar levels of artifacts in CBCT images. On the other hand, Silva et al. ^13^ reported that the radiopacity of a sealer was insufficient to exert any influence on the diagnosis of VRF. In the present study, low values of sensitivity and high values of specificity for VRF diagnosis were found, which indicates that image artifacts produced by endodontic sealers may hinder the diagnosis of fractures.

Dutra et al. ^2^ found that the presence of non-cemented gutta-percha generated a low beam hardening artifact that did not hinder the VRF extension, while Pinto et al. ^12^ reports that hyperdense streak-like artifacts generated by non-cemented gutta-percha impaired the performance in the diagnosis of fractures. Since prior studies assessed the influence of non-cemented gutta-percha in the production of artifacts, the present research added a new approach regarding endodontic sealers. The choice of materials for the current investigation was intended to cover different compositions considering biocompatibility, better adhesion to dentin, and resistance to fracture characteristics. These sealers produce artifacts with different densities according to the proportion of radiopacifiers and other substances in their formulations, such as zinc oxide, bismuth subcarbonate, bismuth oxide, and barium sulfate ^13^ ^,^ ^25^.

Brito-Junior et al. ^25^ analyzed artifacts produced by various endodontic sealers, including AH Plus and Sealer 26, on CBCT images with variations in voxel size, and demonstrated that the endodontic Sealer 26 induced significantly more artifacts than AH Plus in 0.20 mm voxel size image. The author hypothesized that sealers with bismuth oxide, a very radio-dense material, produce more artifacts than sealers with calcium tungstate and zirconium oxide in their formulations, like AH Plus. In agreement with Brito-Junior et al. ^25^, the present study also observed this possible hypothesis, but these results were not significant. Our protocol used a voxel size (0.15 mm) in order to maintain a standard, as in vitro studies showed that this voxel size improves the accuracy of the diagnosis of root fractures in filled teeth and reduces the presence of artifacts ^6^ ^,^ ^7^ ^,^ ^9^ ^,^ ^22^. Celikten et al. ^23^ evaluated the same sealers and found similar results, but also showed that bioceramic sealers cause artifacts in a smaller extension than AH Plus. However, our investigation revealed that all studied sealers had a negative influence in the diagnosis and visualization of VRFs (Table 3). Nonetheless, the bioceramic sealer (BC Sealer) induced significantly more artifacts and presented darker and hypodense images than the other sealers. In addition, image artifacts generated by different sealers impair the diagnosis of VRFs by decreasing sensitivity to distinct degrees.

A recent publication assessed the expression and direction of artifacts caused by metal posts in the anterior and posterior regions of teeth insertion in the mandible ^24^. The authors observed significantly lower mean values of gray in the posterior region of the mandible when compared with the anterior region of the mandible. Accordingly, the present work evaluated the teeth in the mandibular model and the indexes of sensitivity and specificity for VRFs diagnosis were lower in the premolar teeth relative to central incisors.

The limitations of these study occur due to the size of the lesion and the artifacts production, which have already been demonstrated in other in vitro studies ^6^ ^,^ ^7^ ^,^ ^9^ ^,^ ^22^. In conclusion, the current study demonstrates that endodontic sealers may interfere with the diagnosis of VRFs decreasing the sensitivity and increasing the specificity of diagnostic tests. Furthermore, BC Sealer induced significantly more artifacts than other sealers, while fractures in the premolars were harder to detect compared to the central incisors. Therefore, this research invites professionals to consider image differences in addition to the pathognomonic factors of the type of fracture in order to assess the prognosis and determine the appropriate treatment for the tooth.
